# The financial toxicity of breast cancer: a systematic mapping of the literature and identification of research challenges

**DOI:** 10.2478/raon-2025-0002

**Published:** 2025-01-04

**Authors:** Ivica Ratosa, Mojca Bavdaz, Petra Dosenovic Bonca, Helena Barbara Zobec Logar, Andraz Perhavec, Marjeta Skubic, Katja Vörös, Ana Mihor, Vesna Zadnik, Tjasa Redek

**Affiliations:** 1 Division of Radiotherapy, Institute of Oncology Ljubljana, Ljubljana, Slovenia; 2 Faculty of Medicine, University of Ljubljana, Ljubljana, Slovenia; 3 School of Economics and Business, University of Ljubljana, Ljubljana, Slovenia; 4 Sector for Oncology Epidemiology and Cancer Registry, Institute of Oncology Ljubljana, Ljubljana, Slovenia; 5 Sector for Oncology Epidemiology and Cancer Registry, Institute of Oncology Ljubljana, Ljubljana, Slovenia; 6 Faculty of Health Sciences, University of Primorska, Izola, Slovenia

**Keywords:** breast cancer, financial toxicity, bibliometric analysis

## Abstract

**Background:**

Breast cancer is one of the most common cancers, increasingly prevalent also among working-age populations. Regardless of age, breast cancer has significant direct and indirect costs on the individuals, families and society. The aim of the research was to provide a comprehensive bibliometric analysis of the financial toxicity of breast cancer, to identify research voids and future research challenges.

**Materials and methods:**

The systematic mapping of literature relied on a multi-method approach, combining bibliometric methods with a standard review/discussion of most important contributions. The analysis employed Bibliometrics in R and VosViewer.

**Results:**

The results highlighted the key authors, journals and research topics in the investigation of the financial toxicity of cancer and stressed the concentration of work around several authors and journals.

**Conclusions:**

The results also revealed a lack of a comprehensive approach in the study of financial toxicity, as the literature often focuses on one or few selected aspects of financial toxicity. In addition, geographic coverage is uneven and differences in the healthcare systems represent a challenge to straightforward comparisons.

## Introduction

Cancer care is not only a medical challenge, but also a complex socio-economic issue. The term financial toxicity has gained prominence in recent years to describe the adverse financial effects experienced by cancer patients as they navigate diagnosis, treatment, and survivorship.^1^ Financial toxicity in cancer care is prevalent and causes significant financial loss, psychological distress, and maladaptive coping strategies, requiring multilevel, coordinated efforts among stakeholders.^2^ Patients with breast cancer frequently experience financial toxicity as a result of extended and multimodal treatment; in low- and middle-income countries, this was reported to affect 78.8% of patients, while in high-income countries, it affected 35.3% of patients.^3^ Systematic reviews of the literature have shown that patients with cancer from various income-group countries experience a significant financial burden during their treatment^4^, and despite publicly funded universal public healthcare, financial toxicity remains a concern for patients with cancer and their families.^5^ However, patients with cancer in countries with more market-driven health care face more financial toxicity since they have to co-pay for medical services and medicines, even if they have insurance. This is one of the reasons why the prevalence of financial toxicity is higher in the US compared to nations with universal health care (22–27%).^5,6^ Although financial toxicity levels vary by country, the data indicate that financial protection is inadequate in many countries and highlight the need for targeted interventions to alleviate financial strain among affected individuals.^5^ Generally, women fare worse financially than men after cancer treatment.^7^

Various factors contribute to the development and exacerbation of financial toxicity among patients with breast cancer. Socioeconomic factors, such as income level, employment status, and education, play a significant role in determining an individual’s vulnerability to financial strain. Additionally, clinical factors, such as disease stage, further compound the financial burden experienced by patients. Geospatial differences also exist, with certain counties exhibiting higher risk profiles for financial toxicity due to disparities in healthcare infrastructure and access to supportive resources.^7–9^

In a single-institution cross-sectional survey of adult female patients with breast cancer who underwent lumpectomy or mastectomy, lower financial distress was associated with factors such as having supplemental insurance, higher household income, and a higher credit score, while work reduction, increased out-of-pocket spending, advanced tumour stage, and being employed at diagnosis were associated with increased distress.^10^ For survivors of breast and gynaecologic cancer, greater financial toxicity is associated with greater distress and a lower quality of life.^11^

As the incidence and prevalence of breast cancer continues to rise worldwide^12^, understanding its impact on financial toxicity in Europe is essential for guiding policy interventions and improving patient outcomes. In light of these challenges, there is a growing recognition of the need to address financial toxicity as an integral component of comprehensive cancer care. In recent years, the utilization of visualization analysis has surged as a prominent approach for scrutinizing vast bibliometric datasets and results of scientific contributions. This methodology employs specialized software to conduct correlations within data, translating findings into visual representations that facilitate a more intuitive comprehension of pertinent information. By doing so, it facilitates the detection of underlying patterns concealed within extensive datasets, streamlining the assimilation of valuable insights.^13^ While existing literature has comprehensively summarized various aspects of financial toxicity^3,5,13^, there remains a notable need for bibliometric and visual studies examining the current landscape of financial toxicity in patients with breast cancer. Therefore, the aim of present study was to gain insights into the current literature and trends on financial toxicity in patients with breast cancer using bibliometrics and visualization analysis to identify key journals, countries, researchers, institutions, and collaborations among them to identify research voids and future research challenges and discuss most important contributions.

## Materials and methods

### Research goals

This paper relies on a multi-method approach to identify research challenges in the field of the financial toxicity of breast cancer, primarily relying on bibliometric analysis with text mining to provide a solid base for a classic problem-based literature review. The research goal of the bibliometric analysis of the research done within the field of financial toxicity of breast cancer focuses on identifying key challenges and research gaps in understanding the causal relationships between breast cancer, its treatment and direct and indirect financial burden. To do so, the following research questions were addressed:

1. What was the evolution of research in this topic and its dynamics throughout time?
2. Which were the important journals and influential authors who have contributed to the understanding of financial toxicity in cancer, as well as what was the influence of collaboration between authors and countries?
3. Which were the main topics that were investigated in relation to the financial toxicity of cancer? and
4. Which are the current gaps in the literature?
5. While the first two research questions are predominantly explored using bibliometric analysis, the last two are explored using a multi-method approach: the bibliometric analysis is used to provide the general guidelines for further research using content analysis and extended by a standard review of key contributions.

## Methodology

Scopus was used as a base for the bibliometric analysis due to its wide coverage in the field of medicine (including Medline) as well as wider coverage of publication types than Web of Science.*

**FIGURE 1.**
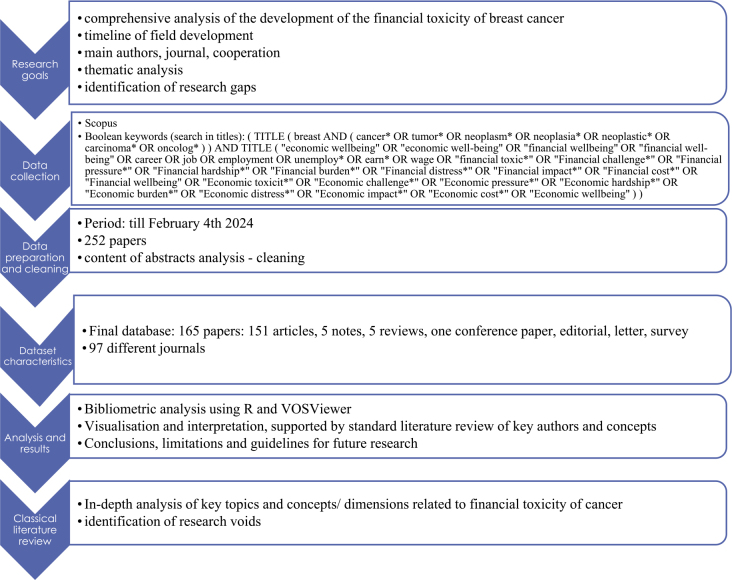
Step-by-step research approach summary (based on ref.^16^).

Initially, 252 papers were obtained from the Scopus database on February 4^th^ 2024, using the search focusing on a wider span of relevant keywords in paper titles (see Figure 1). The final data base was prepared based on content analysis of the paper titles and abstracts to limit the analysis only to those relevant for the study. The final set of studied papers comprised 165 papers (151 articles, 5 notes, 5 reviews, one conference paper, one editorial, one letter, one survey), published in 97 different sources between 1995 and 2024. The papers were prepared by in total 926 authors, with an average of 6.76 authors per paper and only 9 papers being single authored. The content was summarized in 293 different keywords and 1065 key-words plus. The research, presented in the investigated papers, relied on a broad set of knowledge, the total number of cited references was 5323. The investigated body of literature already made a significant impact in the field, since the studied papers were on average cited close to 23 times. Figure 1 summarizes the research approach summary.

Methodologically, the paper combines two approaches: (1) bibliometric analysis, serving as a base for a ^2^ more detailed review of the key literature, identified by the bibliometric analysis. The bibliometric analysis relies on the established bibliometric approaches.^17–20^ The analysis provides first the dynamics of the field development, including key authors, outlets, citation and collaboration. Co-citation and collaboration analyses were used to further explore the relationships between papers, clusters of papers with common topics or origin and also to identify the teams of authors, collaborations that contributed most to the development of the field. The more general thematic analysis was conducted in Bibliometrix package in R online environment (R-Studio 0.98.1091 software).^21^ It was used to extract key topics using keywords and also identify the topics using keyword co-occurrences. Namely, key-words are according to the literature the first and most general summary of the main topics in the text.^22–25^ To further investigate the evolution of themes in the field, a conceptual structure was created using the Multiple Correspondence Analysis (MCA), an exploratory multivariate technique that identifies themes based on distances.^18^ Content analysis relying on keywords was conducted also in R.^26^ The research also utilized VOSviewer (version 1.6.20) for visualization.^27^

## Results

**FIGURE 2.**
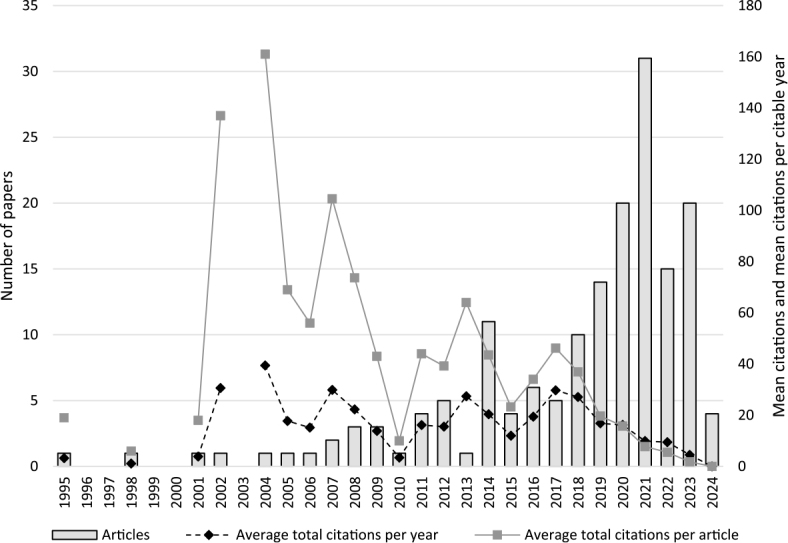
Published number of papers by year (left axis) and mean citations per paper and mean citations per citable year (right axis).

The interest in the topic of financial toxicity of cancer in the literature (focusing on Scopus) has been growing since the 1990s, with the number of papers increasing fast in particular after 2010. In 2021, 31 papers, dealing with the topic of financial toxicity of breast cancer were published. The published papers were on average cited more than 20 times over the observed period. In some years, though, the number of citations in the investigated body of the literature on average exceeded 160 in 2004, 137 in 2002 and 100 citations in 2007, when also some of the more cited papers were published.^28,29^ But, even if the total number of citable years is considered, the investigated body of literature on average still received several citations, apart from the papers published in 2024 (Figure 2).

The outlets, that published most papers on the topic, are the following: *Supportive Care in Cancer*, with 20 papers studying financial toxicity of cancer, followed by *Cancer* (10 papers*), Breast Cancer Research and Treatment* (6), *Journal of Cancer Survivorship* (6), *Psycho-oncology* (5). Bradford law states that there are only a few very productive publications, and a much larger number of those of low(er) relevance. The so-called Zone 1 or core journals are those most often cited in the literature for a specific field and thus most important. Mathematically, the rank is inverse with a proportion of the articles in the journal using a logarithmic scale.^30^

**FIGURE 3.**
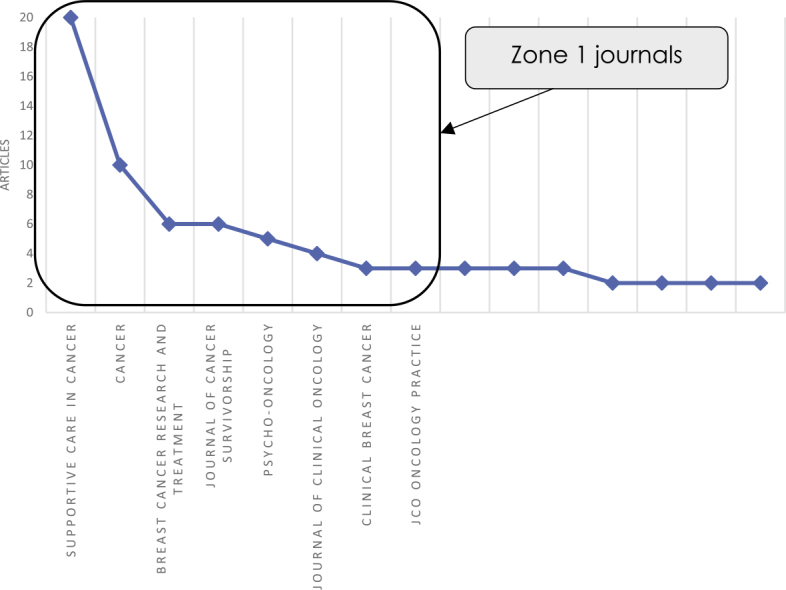
Bradford law with Zone 1 journals.

The Bradford law analysis of the investigated body of literature suggests that the most important sources are indeed *Supportive Care in Cancer, Cancer, Breast Cancer Research and Treatment, Journal of Cancer Survivorship, Psycho-oncology*, but also *Journal of Clinical Oncology, Clinical Breast Cancer* and *JCO Oncology Practice*, which all are in Zone 1 (or most important journals) (Figure 3).

Hawley^31–33^, Offodile^10,34,35^, Wheeler^36–38^, Bradley^29,39,40^ and Jagsi^31,32,41^ are some of the most important authors, authoring or co-authoring at least 6 published articles or 3.6% or more of the investigated body of literature (Table 1, for each author the citations in the brackets in the text refer to the 3 most cited papers).

**TABLE 1.** A list of authors with at least 4 published papers in the investigated set of literature

Authors	Articles	Articles Fractionalized*	No of documents (in % of all)
HAWLEY ST^31–33^	7	0.919	4.2
OFFODILE AC^10,34,35^	7	0.774	4.2
WHEELER SB^36,38^	7	0.868	4.2
BRADLEY CJ^29,39,40^	6	1.569	3.6
JAGSI R^31,32,41^	6	0.701	3.6
ASAAD M^10^,^34^,^35^	5	0.549	3
BOUKOVALAS S^10,34,35^	5	0.549	3
KATZ SJ^31–33^	5	0.576	3
AZUERO A^46^	4	0.522	2.4
CHAN A^64–67^	4	0.342	2.4
COOPER B^64–67^	4	0.342	2.4
GORDON L^45^	4	0.501	2.4
HAMILTON AS^31,32^	4	0.476	2.4
KOCZWARA B^64–67^	4	0.342	2.4
MIASKOWSKI C^64–67^	4	0.342	2.4

* Fractionalized authorship to papers assesses individual productivity taking into account coauthorships, assuming equal distribution of contributions across all authors

Lotka Law^42^, which investigates the concentration (or distribution of papers by authorship) also highlights that 3 authors (Hawley, Offodile, Wheeler), who in total represent 0.3% of all authors, have contributed a significant proportion of the studied papers, while on the other hand 87% of authors have only contributed one paper. H index^43^, measuring authors’ local impact, shows that Hawley, Jagsi, Wheeler and Offodile have the highest H-index of 6, indicating that each has at least 6 papers, each cited at least 6 times.

Authors are international, coming from a number of different countries, most often collaborating with the US (25 papers), UK (13), Switzerland (7), Australia (6). While authors are from a number of different institutions, the most common affiliations are: University of Michigan, University of Texas (MD Anderson Cancer Center), University of California, University of North Carolina, Shiraz University of Medical Sciences, Johns Hopkins University, University of Maryland and Harvard Medical School, each with at least 13 mentions with Michigan in total with 38. USA dominates also among the cited references, with in total 2565 cited papers, followed by Australia with 233, Canada with 180 papers and UK with 145 cited papers in the list of references. Further investigation of the collaboration between groups of authors shows that there are five strong groups of authors, who collaborate frequently. Among those are: (1) Wheeler, Spencer, Blinder, Reeder-Hayes, Swanberg and Vanderpool, (2) Hawley, Bradley, Jagsi, Katz, Hamilton, Abrahamse, Griggs, Janz, Kurian, Wallner, Blinder, and (3) Offodile, Asaad, Boukovalas, Greenup, Lin, Bailey, and Butler, to list just the first three groups of authors.

The investigation of the financial toxicity of breast cancer was highly influenced by a smaller set of highly cited papers (Table 2).

**TABLE 2.** A list of 10 most cited papers in the investigated set of literature (only the first author is listed in case of multiple authors)*

Paper	DOI/PMID	Total citations	TC per year	Normalized TC
Jagsi *et al*., 2014, J Clin Oncol^31^	10.1200/JCO.2013.53.0956	206	18.73	4.73
Arozullah *et al*., 2004, J Support Oncol^28^	PMID: 15328826	161	7.67	1.00
Bradley *et al*., 2002, J Health Econ^29^	10.1016/S0167-6296(02)00059-0	137	5.96	1.00
Bradley *et al*., 2007, Cancer Invest^39^	10.1080/07357900601130664	117	6.50	1.12
Lauzier *et al*., 2008, J Natl Cancer Inst^44^	10.1093/jnci/djn028	111	6.53	1.51
Jagsi *et al*., 2018, Cancer^32^	10.1002/cncr.31532	104	14.86	2.82
Meneses *et al*., 2012, Gynecol Oncol^46^	10.1016/j.ygyno.2011.11.038	94	7.23	2.40
Gordon L *et al*, 2007, Psycho-Oncology^45^	10.1002/pon.1182	92	5.11	0.88
Greenup *et al*, 2019, J Oncol Pract^37^	10.1200/JOP.18.00796	81	13.50	4.12
Wheeler *et al*., 2018, J Clin Oncol^36^	10.1200/JCO.2017.77.6310	81	11.57	2.20

* TC per year = total citations per year; Normalized TC = Normalized total citations

Below, a summary of the most cited is provided. Jagsi and co-authors^31^ published in 2014 the most cited paper with in total 206 citations. They used a longitudinal approach to study the long-term financial burden of breast cancer that showed that a quarter of women suffered financial decline due to breast cancer, and that the minorities were more vulnerable to the effects. Arozullah *et al*.^28^ showed that the financial burden of cancer in the US accounted for at least 26% of monthly income to as much as 98%, depending on income group, and that the insurance policies covered on average only around 3% of out-of-pocket expenditures of the studied women, providing valuable policy input that affordable compensation plans should be available in particular to those in low income brackets. Bradley *et al*.^29^ in 2007 investigated the relationship between breast cancer survival, work and earnings in the US and found that while breast cancer does have a negative impact on employment, the responses of women are heterogenous and that the survivors who do work in fact worked and earned more than those in the control group. In 2002, Bradley *et al*.^39^ showed in a US-based longitudinal study that the greatest impact on labour supply was present in the first six months after diagnosis, while between 12 and 18 months after diagnosis many already returned to work. Among the papers with more than 100 citations is also the work of Lauzier *et al*.^44^ who showed that in Canada on average around a quarter of projected annual wage was lost due to breast cancer, more among those with lower education, those with lower social support, receiving chemotherapy, self-employed and short work-experience, to list just those with highest significance. A longitudinal study in Australia showed that economic costs continue to affect women even 1.5 years after surgery, where income loss and the costs of health service were the most important sources of economic burden, which is higher for women with positive lymph nodes.^45^ Related to the longer-term analysis of financial toxicity of cancer, a follow-up study of 132 survivors showed that the impacts in the longer term are significant in the financial sense (e.g. increased insurance premiums) and otherwise (lower motivation, productivity, quality of work, impact on absence from work), stressing the extended impact of cancer burden on post-treatment period in the US.^46^ Wheeler *et al*.^36^ discuss the racial differences in breast cancer financial toxicity in the US and find that the impact of race was significant for job loss, transportation barriers, income loss, and overall financial impact. Jagsi *et al*.^32^ investigated the role of clinicians’ engagement in the patient care also from the perspective of financial toxicity of cancer, not just health aspects of the disease in the US. Between 15-30% of patients, depending on ethnicity, expressed desire to discuss also financial burden of cancer, however, depending on the topic, between 50 and 70% of those longing to talk also about the financial aspect, did not report or receive such support. Financial toxicity impacts also the decision for the type of breast cancer surgery. For example, more than a quarter of studied women in the US reported that costs were considered when deciding about preservation and appearance.^37^ Bilateral mastectomy was associated with higher debt, very high financial burden and changed employment.^37^ These findings, which refer to the most cited papers, mainly refer to the US, which has a specific health insurance system.

Although the investigated literature focuses on the financial toxicity of breast cancer, the literature deals with a wide array of subtopics. The simplest content analysis is done using keywords, as they are used to efficiently summarize the text.^47^ Most common author-used keywords by frequency are the following: breast cancer and financial toxicity, return to work, quality of life, survivorship, cost of illness, costs, metastatic breast cancer, oncology, cancer survivors/survivorship, chemotherapy, financial burden, lymphedema, fatigue, healthcare costs, treatment, financial stress, occupation, rehabilitation, social support, unemployment, work.

Thematic map, investigating the relationships between the words, prepared in Bibliometrix, allows the division of the topic also into basic themes, motor themes, niche themes and emerging/declining themes, which are investigated using keywords for each theme (100 words were included, minimum cluster frequency 5, Walktrap clustering algorithm). Table 3 summarizes the main topics and provides selected references for each of the identified topics.

**TABLE 3.** Thematic map of (financial) toxicity of breast cancer with most common author keywords for each of the themes and selected references

	Key term(s)	Other key terms*	Selected papers (No. of reference)
	T1: Breast cancer (neoplasms), employment, financial toxicity	Quality of life, return to work, (cancer) survivor(ship), treatment, financial burden / stress, fatigue, chemotherapy, mental health, caregivers, social support, disability, occupation, burnout complaints	29, 33, 39
**Motor themes**	T2: Metastatic breast cancer	Prevalence, healthcare use, healthcare utilization, healthcare costs, advanced breast cancer, adverse effects, administrative claims, breast cancer costs	48, 49
	T3: COVID-19	Depression, job loss, access to healthcare, breast cancer survivors, cognition, anxiety, autonomy	50, 51
**Basic themes**	T1 Economic burden	Cost(s), oncology, lymphedema, rehabilitation, breast neoplasm, cost-effectiveness, recurrence, screening, cost of illness, cancer, resource utilization, healthcare use	52–54
**Emerging or declining themes**	T1: Coping strategies	Breast cancer, healthcare, costs, regional, ethnic differences	55, 56
**Niche themes**	T1: Reasonable accommodations	Sick leave, assessment and planning	57, 58

* Other key terms (T) selected based on centrality and repetition (overlap with other similar key terms within same topic).

The **motor themes** are three (T1–T3, Table 3). The first motor topic is related to the individual and the consequences of the **disease** for the individual, in particular **in relation to employment and financial toxicity**. This topic deals with cancer survivorship, employment and the return to work, occupational differences, related disability and the consequences of treatment (chemotherapy, fatigue, burnout) as well as mental health aspects of the disease. In terms of financial toxicity, a number of aspects are investigated, besides employment also unemployment, social support, rehabilitation, return to work, occupations, needs assessment (which can also be related to return to work), socio-economic status, sick-leave, career change, fatigue, job loss, quality of life, and other.^29,33,39^ The second motor topic is related to metastatic breast cancer, its prevalence, the impact on healthcare use, utilization and costs. This topic is more closely related to the wider healthcare aspect of cancer-related cost.^48,49^ The third motor topic was dealing with COVID-19 and breast cancer.^50,51^ The COVID-19 is on the margin between a niche and a motor theme, indicating a fast development of a narrow theme, which focuses on the impact of cancer during COVID-19, to job-loss and mental health. The access to healthcare was also highlighted. The topic of financial toxicity of cancer (T1, Table 3) is also close to the border between basic and motor themes, while the broader economic burden is a major motor theme. General economic burden, cost of illness and cancer is a **basic theme**. The key words stress the cost-effectiveness, resource utilization, healthcare use and healthcare costs, screening. The topic also highlights differences between diagnoses (e.g. metastatic, hormone positive, premenopausal). A close link between the motor theme (T1) and basic theme is for example productivity loss, which highlights the aggregate effects of the impact of the disease on the individuals’ labour market outcome.^52–54^ **Niche themes** revolve around reasonable accommodations and sick-leave, highlighting also the importance of assessment and planning.^55,56^ **Emerging or declining themes** revolve around coping strategies, healthcare costs as well as regional and ethnic differences.^57,58^

An investigation into the evolution of the themes between 1995 and 2024 shows that before 2010, the number and diversity of the topics in the literature was significantly narrower, focusing primarily on (1) employment (hours worked, labour market effects, disability, earnings), (2) process of treatment and return to work (oncology, breast neoplasms, chemotherapy, rehabilitation, occupation, return to work), (3) process of the return to work (assessment and planning, reasonable accommodations, job retention), (4) healthcare system and costs (prevalence, direct and indirect costs, cost of illness, administrative claims) and (5) selected demographic aspects. After 2010, the number of topics significantly increased, predominantly due to further disaggregation of selected aspects. In addition to the aforementioned key aspects, which were driving the literature before 2010, several additional aspects emerge: (1) financial toxicity in relation to coping strategies, social supports, community programs, (2) metastatic cancer is studied in relation to cancer distress, costs, role of screening, (3) healthcare costs and use are studied in relation to cost drivers and adverse events, while also (4) covid-19 emerges as a topic, both in relation to financial toxicity as well as anxiety, and cognition.

## Discussion

### Discussion of bibliometric analysis: a review of most important findings

According to the results of the bibliometric analysis, the literature on the financial toxicity of breast cancer is marked with a significant concentration in terms of relevant research journals (*Supportive Care in Cancer, Cancer, Breast Cancer Research and Treatment, Journal of Cancer Survivorship*), authors (Hawley^31–33^, Offodile^10,34,35^, Wheeler^36–38^, Bradley^29,39,40^ and Jagsi^31,32,41^) as well as concentration of topics, with the two most important and widest being the (1) individual-level investigation of financial toxicity of cancer in relation to earnings, employment and other related topics and (2) a more aggregated health-care and social system perspective related to cancer treatment and its costs.

However, the financial toxicity of cancer is a much wider concept, encompassing (i) direct or active financial spending, (ii) passive financial resources’ spending, (iii) psychosocial impacts, (iv) the need for external support, (v) coping with care and (vi) changes in lifestyle.^47^ The direct payments include medical (potential treatment expenses, hospitalization expenses, depending on social security system), non-medical costs (travel, accommodation, other travel related costs), out-of-pocket costs (medications, deductibles and co-payments, depending on social system).^59,60^ Second, the individual suffers loss of income due to reduced working hours or even job-loss^61^, domestic finances and assets can be affected due to the use of savings^62^, and individuals can suffer insurance-related costs (increased premiums).^63^ In the short and in the long-term, the disease can bear significant costs due to stress and anxiety, while the quality of life can also suffer. In the long term, primarily the so-called survivorship costs, related to on-going care or long-term effects of cancer and potential recurrence are important.

The investigated body of literature, which examines financial toxicity of breast cancer, focuses most on the employment, job, and income related consequences (Table 4).

**TABLE 4.** A systematization of (financial) toxicity of breast cancer at the level of the individual (left column) and research gap (right column)

Type of financial burden/burden	Coverage in the literature and research gap
1. Medical costs	Weaker coverage, survey based, depends on social security system, more relevant for private-insurance based system (e.g. US)
Treatment expenses	
Hospitalization costs	
2. Non-medical costs	Weaker coverage, survey based, depends on social security system, more relevant for private-insurance based system (e.g. US)
Travel expenses	
Accommodation costs	
Other	
3. Out-of-pocket costs	Weak coverage, survey based
Deductibles and co-payments	
Prescription drug costs*	
4. Loss of income	Well-documented employment impacts, income impacts, less focus on occupational change
Changed work hours	
Job loss	
Change in occupation	
Loss/change in income	
5. Insurance-related costs	Weak coverage, depends on social security system, but has broader relevance for other non-medical insurances (life, travel, etc.)
6. Impact on finances and assets:	Weak coverage
Debt accumulation	
Asset depletion	
7. Psychosocial impact: Stress and anxiety	Well-documented, focus on stress, anxiety, less focus on quality of life as a whole
Stress and anxiety	
Quality of life	
8. Long-term financial consequences	Increasing interest on recurrence, screening
Survivorship costs	
Cancer recurrence	
9. Geographical coverage	Vast body of evidence for the US, poorer coverage for EU/European context
US	
Europe	

* Can differ between countries depending on health-care system

These are also the consequences that can more easily and reliably be measured, either via surveys or registry-data, both cross-sectional and longitudinal, focusing also on the differences conditional on the demographic characteristics of patients. The literature also demonstrates a lot of focus on phycological impacts on the individual, which can have longer-term effects on both health as well as financial stress. The aggregated perspective on the health-care system is also at the forefront of research. On the other hand, the reviewed body of literature on financial toxicity of breast cancer displayed little interest in the non-medical costs, insurance related costs, impact on debt accumulation and depletion of savings. However, crosscountry differences are notable, depending not only on the health-care system, but also on the income (development) level of the countries.^4,68^ In particular, when comparing developed economies, the evidence is widely focused on the experiences of the US patients, there is significantly less evidence for European context.^69–72^ The studies show a significant level of financial burden of cancer in both US and EU, however, in the US the private insurance, varying insurance coverage and reimbursement policies referring to cancer care, including diagnostics, treatments (chemotherapy and radiation), medications and also supportive care medications cause substantial out-of-pocket expenses for patients.^60^ In Europe, where healthcare systems are predominantly publicly funded and universal, breast cancer patients generally face lower out-of-pocket costs for medical services. However, disparities in access to innovative treatments and supportive care services may still exist across different European countries, contributing to variations in financial toxicity among patients^4,73,74^, which highlights also the need for using an adjusted methodology.^75^

### Limitations and future research orientation

This analysis contributes to the literature in several aspects. First, it studies the body of literature on the financial toxicity of breast cancer in Scopus. A comparable analysis using Web of Science^76^ is narrower due to the coverage as well as due to its focus on solely bibliometric issues. This paper relies on a multi-method approach to provide a more comprehensive overview – first, it highlights in a systematic manner the most notable authors and papers as well as stresses the concentration of authors, journals and topics in the literature. Second, the paper shows that the majority of the literature focuses on selected aspects of financial toxicity of cancer. Thereby, it identified a research gap that can propel future development of the study area.

The analysis can in the future also be extended and improved to overcome some of the limitations of the existing analysis. First of all, a more detailed analysis into each of the key topics would allow identification of main linkages between the variables of interest within a specific topic. An in-depth investigation of each of these variables would allow identification of possible causal mechanisms in the existing literature that explain the channels through which cancer is related to financial toxicity in both short and long term. It is also important to highlight the methodological downsides of bibliometric analysis^77^, which is in fact quantitative, although it often seeks to provide qualitative conclusions. Furthermore, the body of literature is focusing on different health-care systems, revealing also the differences in the financial toxicity. Future research should adequately address these differences in empirical assessment^75^, in particular when comparing different countries. This could also imply that data gathered based on established international methodology (questionnaires such as Comprehensive Score for financial Toxicity - Functional Assessment of Chronic Illness Therapy (COST-FACIT)^78^ should be used with care and questionnaires should be extended to capture national specifics.

## Conclusions

The financial toxicity of breast cancer represents a burden that encompasses a wide range of effects, from the direct to the indirect financial costs as well as wider socio-economic impacts on patients. This paper provides a systematic mapping of the literature, relying on the bibliometric analysis that shows that despite the relatively wide coverage, there are still significant research gaps in the literature. The literature often concentrates on specific aspects of financial toxicity, is often focusing on one country and thereby also one specific healthcare system, or is not addressing the broader, more holistic aspects of the problem. In particular, the literature is focusing on the aspects that are easier to measure or capture, while a more holistic approach would require both a broader as well more often also a longitudinal approach. Such an approach would also allow better informed policymaking to alleviate the short- and long-term effects of the financial toxicity of breast and other cancers.

## Supplementary Material

Supplementary Material Details

## Footnotes

* For example, in December 2023 Scopus included more than 29200 active serial publications, more than 330 thousand books and 23.4 million open access items from more than 7000 publishers. In total, the data comprised the work of almost 20 million authors, and almost 100 thousand affiliations.^14^ Scopus also includes data from MEDLINE and EMBASE.^15^ The wider and highly topic-relevant coverage were the main reasons why Scopus was chosen over Web of Science.^16^
