# Supplementary material for: The financial toxicity of breast cancer: a systematic mapping of the literature and identification of research challenges
Source: Radiol Oncol. 2025 Jan 4;59(1):31–42. doi: 10.2478/raon-2025-0002 (PMC11867575; doi:10.2478/raon-2025-0002)
Supplement: Supplementary file 1 — Supplementary Material Details [file raon-2025-0002_sm.pdf]

sciendo Radiology and Oncology | Ljubljana | Slovenia | www.radioloncol.com
RADIOLOGY AND ONCOLOGY

Investigation of *GSTP1* and *PTEN* gene polymorphisms and their association with susceptibility to colorectal cancer

Durr-e-Shahwar, Hina Zubair, Muhammad Kashif Raza, Zahid Khan, Lamjed Mansour, Aktar Ali, Muhammad Imran[1]

doi:10.1515/raon-2025-????

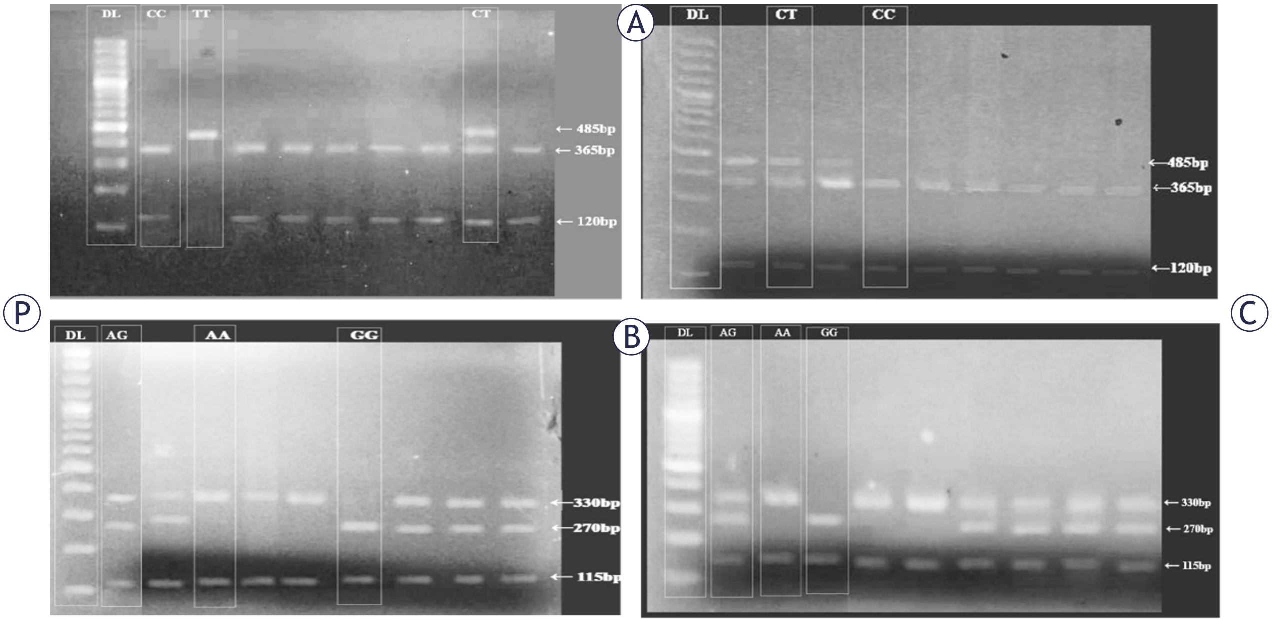

SUPPLEMENTARY FIGURE 1. Representative images of GSTP1 genotyping. **(A)** The C/C genotype of rs1138272 was identified as 120 and 365 bp fragments, C/T as 120, 365 and 485 bp fragments and T/T genotype was identified as 485 bp (AciI) fragment. **(B)** The homozygous A/A genotypes of rs1695 were identified as 115 and 330 bp fragments, A/G as 115,270 and 330 bp fragments while G/G as 115 and 270 bp fragments (Alw261).

DL = DNA ladder; C = Control; P = Patients

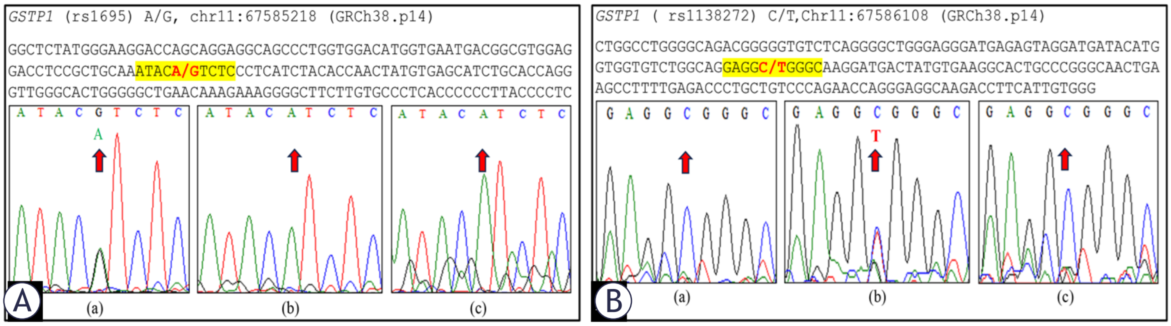

SUPPLEMENTARY FIGURE 2. Sanger sequencing chromatograms for **(A)** *GSTP1* rs1695 (a) GA genotype (b) AA genotype (c) AA genotype; **(B)** *GSTP1* rs1138272 (a) CC genotype (b) CT genotype (c) CC genotype

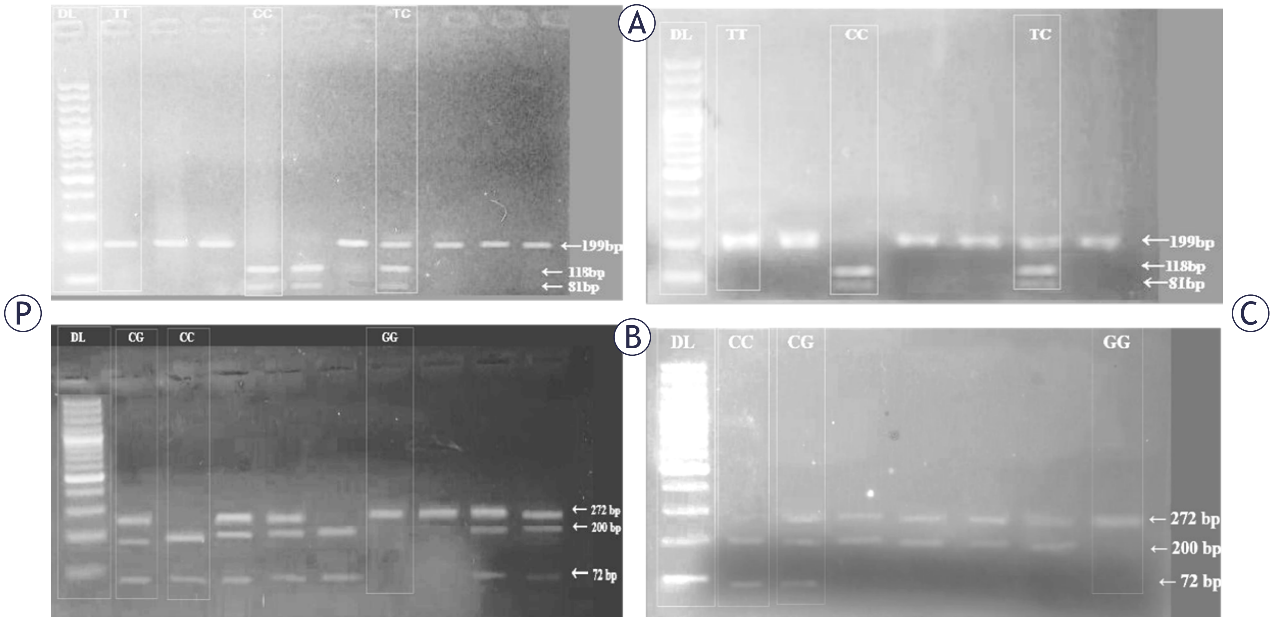

SUPPLEMENTARY FIGURE 3. Representative images of PTEN genotyping. **(A)** The T/T genotype of rs701848 was identified as 199 bp fragment, T/C as 81,118 and 199 bp fragments and C/C genotypes were identified as 81 and 118 fragment (HaeIII). **(B)** The homozygous C/C genotypes of rs2735343 were identified as 72 and 200 bp fragments, C/G as 72, 200, and 272 bp fragments. While the G/G genotype was identified as a 272 bp fragment (HhaI).

DL = DNA ladder; C = Control; P = Patients

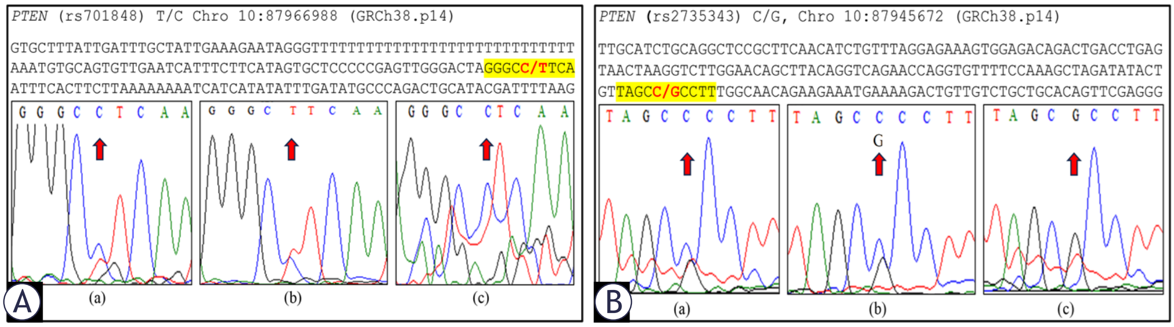

SUPPLEMENTARY FIGURE 4. Sanger sequencing chromatograms for **(A)** *PTEN* rs701848 (a) CT genotype, (b) TT genotype (c) CC genotype, and **(B)** *PTEN* rs2735343 (a) CC genotype, (b) CG genotype, and (c) GG genotype.
